# Patient information leaflets: informing or frightening? A focus group study exploring patients’ emotional reactions and subsequent behavior towards package leaflets of commonly prescribed medications in family practices

**DOI:** 10.1186/1471-2296-15-163

**Published:** 2014-10-02

**Authors:** Oliver Rudolf Herber, Verena Gies, David Schwappach, Petra Thürmann, Stefan Wilm

**Affiliations:** Institute of General Practice, Medical Faculty of the Heinrich Heine University Düsseldorf, Moorenstr. 5, Building 14.97, 40225 Düsseldorf, Germany; School of Nursing & Midwifery, University of Dundee, 11 Airlie Place, Dundee, DD1 4HJ Scotland, UK; Department of Clinical Pharmacology, University of Witten/Herdecke, Witten, Germany; HELIOS Klinikum Krefeld, Hospital Pharmacy, Krefeld, Germany; Swiss Patient Safety Foundation, Zurich, Switzerland; Institute of Social and Preventive Medicine (ISPM), University of Bern, Bern, Switzerland; Philipp Klee Institute for Clinical Pharmacology, HELIOS Klinikum Wuppertal, Wuppertal, Germany

**Keywords:** Patient information leaflets, Medication adherence, Qualitative research, Focus groups, Anxiety, General practitioner, Family practice, Type 2 diabetes, Hypertension, Hypercholesterolemia

## Abstract

**Background:**

The purpose of patient information leaflets (PILs) is to inform patients about the administration, precautions and potential side effects of their prescribed medication. Despite European Commission guidelines aiming at increasing readability and comprehension of PILs little is known about the potential risk information has on patients. This article explores patients’ reactions and subsequent behavior towards risk information conveyed in PILs of commonly prescribed drugs by general practitioners (GPs) for the treatment of Type 2 diabetes, hypertension or hypercholesterolemia; the most frequent cause for consultations in family practices in Germany.

**Methods:**

We conducted six focus groups comprising 35 patients which were recruited in GP practices. Transcripts were read and coded for themes; categories were created by abstracting data and further refined into a coding framework.

**Results:**

Three interrelated categories are presented: (i) The vast amount of side effects and drug interactions commonly described in PILs provoke various emotional reactions in patients which (ii) lead to specific patient behavior of which (iii) consulting the GP for assistance is among the most common. Findings show that current description of potential risk information caused feelings of fear and anxiety in the reader resulting in undesirable behavioral reactions.

**Conclusions:**

Future PILs need to convey potential risk information in a language that is less frightening while retaining the information content required to make informed decisions about the prescribed medication. Thus, during the production process greater emphasis needs to be placed on testing the degree of emotional arousal provoked in patients when reading risk information to allow them to undertake a benefit-risk-assessment of their medication that is based on rational rather than emotional (fearful) reactions.

## Background

A patient information leaflet is a technical document included in every medicine package to offer written information about the medication. Patient information leaflets (PILs) are provided by the manufacturer following a standard template consisting of the same types of information for every medication. Their main purpose is to inform patients about their medication regarding its administration, precautions and potential side effects. As required by Article 11 of Directive 2001/83/EC, the content of PILs ought to be unbiased, evidence-based and presented in a clear, understandable and well-readable way to suit laypersons [1].

In 2004, the amended Directive 2004/27/EC demanded readability and comprehension testing of PILs [2]. The European Commission issued the "Guideline on the readability of the label and package leaflet of medicinal products for human use", which provides guidance on how to produce accessible and understandable package leaflets [3]. More specifically, the guideline provides advice on the presentation of the content, design and layout including guidance on consultations with target patient groups and a way of undertaking a test of a package leaflet in order to optimize its usability. Specific recommendations for blind and partially-sighted patients are also provided. As of November 2005 the European Commission guideline led to the introduction of readability user tests to demonstrate the readability and usefulness of the package leaflet to patients. Such tests became obligatory for newly authorized medicinal products [3, 4].The purpose of user testing is to achieve legible, clear and easy to use package leaflets [3]. Member states and the European Medicines Agency agreed on harmonized Quality Review of Documents (QRD) templates for package leaflets to ensure linguistic clarity, consistency and accuracy of PILs [4, 5].

Despite substantial regulatory efforts to improve readability and comprehensibility patients to date are still confronted with long texts written in small font size [6], non-comprehensible medical terms [7] and poorly presented statistical information leading to misjudgment of adverse effects and consequently poor decisions on the medically prescribed treatment [7, 8]. For example, a German survey found that one quarter of participants were unnerved by the negative information and stopped taking their medications after reading about potential side effects [9]. Similar results were obtained from outside the European Union where over two-thirds of Australian general practitioners (GPs) reported patients refusing to take their medication because of poorly designed PILs [10]. Non-adherence to prescribed medication regimens accounts for substantial worsening of disease, death and increased health care costs [11, 12].

Until now numerous studies have focused on how PILs could be improved with regard to design [13], readability [14] and understanding of adverse effects [7]. Yet little is known about the way patients react when reading risk information conveyed in PILs and their subsequent behavior. This paper reports a subset of findings derived from a larger qualitative study component. Within the scope of this paper we focus on patients’ reactions to PILs which accompany medications commonly prescribed by GPs for the treatment of Type 2 diabetes (DM), hypertension (HT) or hypercholesterolemia (HC). These classes of drugs have been chosen since they are the most widely prescribed [15] and are the most frequent cause for consultations in GP practices in Germany [16].

## Methods

Focus groups were chosen because they are the most appropriate method for different groups of people to interact in order to gain new knowledge and generate meaningful suggestions, opinions and feedback [17]. Furthermore, focus groups provide a means of listening to the perspectives of key stakeholders particularly when current knowledge about a complex phenomenon is insufficient and expansion is vital [18]. As compared to one-to-one interviews, focus groups allow group dynamic processes and permit the observation to observe the extent and nature of participants’ agreement and disagreement regarding the topic under investigation [19].

### Recruitment of participants

The study was approved by the research ethics committee of the University of Witten/Herdecke, North Rhine-Westphalia, Germany. Out of 43 patients recruited by their GPs, 35 (9 women) agreed to participate in six focus groups that were conducted between February and June 2009. Reasons for refusal to participation (4 women, 4 men) included reduced hearing ability, illness of a spouse or extended holidays. GPs were used to recruit participants because they function as gatekeepers in the German health care system. Prior to conducting the focus group discussions 13 GP practices which form part of the universities network of research practices were contacted in writing and invited to recruit patients for the study. Seven of those got involved in the recruitment process. Participating patients were selected by their GPs according to predefined criteria. Patients were eligible if: (1) they had been prescribed oral medication for at least one of the aforementioned conditions and (2) were able to speak and understand German. Patients’ contact details were forwarded to the researchers for scheduling the focus groups. Prior to the focus group discussions, candidates were informed of the goal of the focus group. Gender imbalance of participating patients may be attributable to the selection of the GPs. All participants signed informed consent forms before the focus group and were reassured that their identities would remain confidential. Each participant received €40 to cover incidental and travel expenses, thus ensuring that costs did not affect participation.

### Focus groups and generating data

The six focus groups were composed of five to seven patients [20]. The focus group discussions took place in a youth center in Herdecke – about 7 miles away from the university; a central venue convenient to all participants and a neutral meeting point. Small refreshments were served to create a relaxed atmosphere. All focus groups were conducted in German. A focus group discussion guide was developed using the procedure described by Kruse [21] and pilot-tested to ensure clarity in wording of questions. Participants were asked in advance to bring along PILs of their current medication to stimulate discussion. Two researchers (ORH, VG) were present at each focus group; one facilitated the discussion, the other took detailed notes of the main points of the discussion. In order to reduce peer-pressure the trained interviewer – who was not known to the participants – took on a moderator’s role clarifying that there are no right or wrong answers. The following broad question was asked: "You are currently taking tablets and in each medicine box there is a patient information leaflet. Try to remember last time your GP prescribed you a new medication. What did you think about the patient information leaflet that was enclosed with the medication?" Additional more specific questions were asked in the course of the discussion (see Table 1 for focus group discussion guide). All focus group discussions were recorded digitally and transcribed verbatim. Focus groups were conducted until theoretical saturation was reached, i.e. no new themes appeared [22]. Please note that all quotes used below are translations from German into English.

**Table 1:** **Focus group discussion guide**

Stimuli	Prompts
**1** ^**st**^ **Stimulus**	
"You are currently taking tablets and in each medicine box there is a patient information leaflet. Try to remember last time your GP prescribed you a new medication. What did you think about the patient information leaflet that was enclosed with the medication?"	• What do you think is the purpose of a patient information leaflet?
**2** ^**nd**^ **Stimulus**	
"In your opinion, what should a ‘good’ patient information leaflet look like?"	• What do you like most about your patient information leaflet?
	• What is it you like least about your patient information leaflet?
	• Did you ever have problems with your patient information leaflet? Can you tell me about that?
**3** ^**rd**^ **Stimulus**	
"If you were given the opportunity to design a patient information leaflet, what would you include?"	• Are you able to find your bearings in the patient information leaflet?
**4** ^**th**^ **Stimulus**	
"What thoughts/feelings do you have when reading your patient information leaflet?"	• Please try to remember your thoughts or feelings when reading a patient information leaflet and describe them in as much detail as possible.
**5** ^**th**^ **Stimulus**	
"Are there any parts in your patient information leaflet that you don’t understand?"	• What kind of support do you need in order to understand the content of your patient information leaflet?
"If so, which ones are those?"	• Do you use any other sources of information apart from the patient information leaflet?
**Finally: Coming to a close**	
"Is there anything that you would like to add or mention?"	

### Data analysis

Data analysis was carried out parallel to conducting the focus group discussions. The analysis of the transcribed focus group discussions was performed by a multidisciplinary team consisting of a pharmacist, two GPs, a nurse researcher and two patient representatives not involved in the study to mirror the patients’ perspective (analysis team). None of the coders was affected by any of the three conditions under discussion in this context. In order to ensure internal consistency of coding, one team member assumed overall responsibility for analyzing the data [23]. The analytic process was guided by thematic framework analysis [24]. The following analysis procedure was utilized. Firstly, during the familiarization process all transcripts were read several times to identify recurring themes or ideas. Secondly, an initial coding framework was developed by two coders through establishing themes and sub-themes; relationships between them were identified. The third step involved coding of data according to the initial coding framework and amendment of the framework. During this process half of the interview transcripts were read and coded for themes independently by each coder and then the coding was undertaken jointly by the entire analysis team to ensure coherence; a line of action that increases the credibility of the findings. This process led to the refinement of the coding framework by adding or amending themes and sub-themes. We then applied this final coding framework to all interview transcripts. Finally, descriptive and explanatory accounts were developed. The analysis was carried out using the software package MAX-QDA to manage the data. In establishing trustworthiness, the researchers provided feedback to the participants to ensure credibility of the data [25]. To this end, participants were invited to the university to review and comment on the categories. The majority of themes were confirmed; minor discrepancies were clarified and resolved through discussion.

## Results

The mean age of participating patients was 64.5 years with the majority aged 50 or above; their age ranged from 38 to 79 years. While most participants were of German origin, three had a migrant background (Turkish, Belgian). Most participants were diagnosed with at least two of the diseases that rendered them suitable for inclusion in the study. The mean number of regular daily tablets to be taken was 6.5 (range: 1 to 21). The average duration of the focus group discussion was 123 minutes with a range of 96 to 150 minutes. During the focus groups participants engaged well with the topic. From the wealth of material that our focus group discussions have generated the following results will focus on a specifically selected subset of data pertinent to patients’ emotional reactions and their subsequent behavior caused by PILs. Three categories will be presented: (i) The vast amount of side effects and drug interactions described in PILs provoke various emotional reactions in patients which (ii) lead to specific patient behaviors of which (iii) consulting the GP for assistance is among the most common.

### Emotional reactions caused by PILs

Possible side effects and drug interactions were among the most prominent issues discussed across all focus groups. Several discussions revolved around the general question of whether side effects should be included in PILs. The vast majority of participants, however, considered such information important enough to be included. Some participants spoke in favor of including only what they called ‘most severe’, ‘relevant’ or ‘important’ side effects without being able to specify these attributes further. The majority of participants agreed with the terminology for frequency of occurrence of side effects suggested by the European Commission Pharmaceuticals Committee and wanted only ‘very common’ and ‘common’ side effects to be included. ‘Uncommon’, ‘rare’ and ‘very rare’ side effects were deemed expendable as they had the potential of upsetting the reader unnecessarily as the following quote demonstrates.

*"Very common" and perhaps even "common"; more than 1 in 10 users is very common, well, this [side effect] is going to occur more frequently. Ok, "common" perhaps as well; this is 1 in 100 users but as far as I’m concerned "uncommon" side effects no longer need to be included. And "rare" and "very rare" is merely upsetting* (3/165).

The most frequently reported emotional response triggered by the overall appearance of PILs was "fear". For many participants the appearance of PILs was decisive of whether they were being read. Almost all participants perceived their current PILs as frightening. Patients stressed the importance of designing PILs in a way that they felt encouraged to read them. Especially the side effects described in current PILs frightened participants and caused them a great deal of uncertainty about whether they would be affected by them. Participants used the notion of "being shocked" to describe their feelings triggered by the vast amount of information contained in PILs and the severity of potential side effects. In addition, the use of a much too small and difficult to read font size had a rejecting effect. Some participants felt literally ill when reading the PIL as the following quotes illustrate:

*If I see all the side effects of the drug I am already ill* (5/346).

*Generally, you shouldn’t read the side effects otherwise you might think you are already dead* (4/33).

### Response behaviors triggered by current PILs

During the focus group discussions participants described numerous behaviors which were provoked when reading PILs: (1) they stopped reading the leaflet; (2) they glanced through the leaflet; (3) they discarded the leaflet; (4) they accessed additional information from alternative sources; (5) they sought support from other professional and lay sources; (6) they stopped taking the medication or (7) altered the prescribed dosage without consulting the GP.

Within this key category the two most frequently mentioned response behaviors were: ‘seeking support’ and ‘stopping the medication’. With regard to the former, participants contacted their GPs, pharmacists, family members or friends. This was particularly true for those with a migration background whose command of the German language was perceived as being insufficient. Participants said they were thankful for a knowledgeable person who could explain the content of the PIL in plain language. Accessing additional information from alternative sources such as consulting a medical dictionary or the internet was a frequently employed strategy for clarification. Some participants reached the decision of whether or how to take the medication based on such sources.

Another frequently reported response behavior was taking a break from the prescribed medication. In five out of six focus groups participants reported on discontinuing their medication after reading PILs. The often lengthy and incomprehensible description of possible side effects was a major reason for this decision. In all but one focus group it was mentioned that the huge amount of potential side effects would outweigh the benefits of the medication and therefore patients did not take their drugs or reduced the dosage without consulting their GPs. The following quotes get to the point of this:

*If you read the entire thing [PIL], you would not want to take the tablets anyway* (1/147).

*My wife says: "I’m getting anxious [when reading the PIL], I don’t take the tablets." And I say to her: "Just throw the patient information leaflet away and take the tablets"* (2/317).

Yet another commonly reported response was contacting the GP or less often the pharmacist. Both professional groups were considered to be in a position to provide advice regarding the prescribed drug. Although participants expressed a strong desire to receive customized information from their GP, they did not necessarily consult them at times because they thought that their GP was too busy. Instead pharmacists would be sought out for assistance. However, it was attested that GPs were the ones who had an overview of all the medications a patient was taking. Even if PILs were well written and easy to comprehend they could never substitute for a GP’s individual judgment; PILs were generally seen as less helpful than face-to-face advice.

### The patient-physician relationship

Since PILs were not considered as being able to take the place of a GP’s individual judgment, participants had much confidence in their GPs and believed that they were best positioned to know what was good for them. Thus, participants were convinced that tailored information regarding the medication and its undesirable side effects was best provided by their GPs. Some participants wished that GPs would explain in plain language the medical terminology used in PILs as the quote below highlights. Participants also felt it is vital to have a follow-up appointment when a new medication was prescribed to see if it had the desired effect. Consulting the GP became even more important if side effects occurred; a situation in which a leaflet is regarded as not being useful.

*All these medical terms which patients don’t understand anyway. Each time [I read the PIL] I need a GP as mediator who explains everything to me (6/298)*.

Despite the above, participants mentioned that GPs would sometimes prescribe medication without providing any instructions on how to take the medication. Yet GPs were considered as crucial mediators between the information contained in PILs and their patients. Some participants mentioned that after the prescription of a new drug they were more likely to adhere to it but more reluctant to read the PIL. For participants with a migration background insufficient knowledge of the German language prevented them from reading PILs. Instead, they relied heavily on their GPs to make sense of the information irrespective of the quality of the PIL.

Participants were well aware that some medications should not be combined with others or that the efficacy of a drug could be altered when given in combination with others. Participants trusted their GPs in communicating this relevant information to them. Yet they also criticized that their GPs did not have the time for it or forgot to discuss possible drug interactions. Because of this, participants thought that drug interactions should be included in PILs to provide users with the opportunity to look them up for themselves in order to make informed decisions regarding their prescribed medication.

## Discussion

The findings of our focus groups showed that PILs – despite European Commission guidelines and those of other legislative bodies – still have considerable need for (linguistic) improvements. It has been demonstrated that PILs included in medicine prescribed for the treatment of Type 2 diabetes, hypertension or hypercholesterolemia have a deterrent effect on patients. PILs contained too much risk information which was conveyed in a way that led to reduced patient compliance. Instead of PILs contributing to providing a sense of security or reassurance for patients, they provoked negative emotions which – among other reactions – led participants to discontinuing their medication or altering the dosage without prior consultation of their GPs.

The findings demonstrated that the current description of potential side effects and drug interactions caused negative emotions which led to undesirable patient reactions. This finding is similar to Nink & Schröder [9] who found that 29% of study participants felt insecure when reading PILs. Insecurity towards PILs increased further with advancing age affecting almost 37% of participants aged 60 and above. Similarly, investigations undertaken by Koo *et al.* [26] and Lee *et al.* [27] found that PILs were considered incomprehensible and frightening. Major contributing factors limiting the usefulness of PILs related to their length, legibility, readability, design, comprehensibility and content [27, 28, 14, 29]. Another study analyzing 50 PILs of frequently prescribed medications reported that only very few manufacturers complied with current European guidelines; 40% gave no indication of the likelihood of adverse effects occurring [7]. While the quality of PILs could be improved by considering key linguistic features [30] it has been said that complex organizational politics, goal conflicts and various other pressures involved in creating PILs adversely affect the comprehensibility of the texts for patients [31].

The focus groups also revealed that PILs provoked certain behaviors in patients including accessing information from alternative sources or seeking support from professional and lay persons. Similar patient behavior has been reported by Lee *et al.* [27] where many study respondents were dissatisfied with the quality of the PILs but only a minority sought information from professional and lay sources. Although only a few patients searched the internet or consulted reference books for pharmaceutical information, such sources were regarded as vital alternatives. The study undertaken by Vander Stichele *et al.* [32] investigating attitudes of physicians towards PILs for patients revealed that 92% of physicians thought that such overt information seeking behavior is limited to a minority of patients and not a general feature of their behavior. In this context it is important to note that risk information on pharmaceutical and third party websites is often incomplete or chaotic due to the absence of quality control mechanisms [33] leaving patients ill-informed or even misinformed about known adverse effects [34] and contraindications [35].

There was a strong emphasis in the focus groups that GPs were considered the best source and enjoyed participants’ trust in providing general drug information as well as patient-tailored knowledge about side effects and drug interactions. Even pharmacists were not accepted as an equally competent source of delivering this information. Similar results have been obtained by Lee *et al.* [27] where GPs trusted unquestioningly and were regarded as the primary provider of information about prescribed medications, followed by community pharmacists. This is contrary to the findings by Nair *et al.* [36] who found that most patients consider pharmacists as the primary source of information since access was the main factor in determining from whom information was sought. It is important to note that the majority of respondents in our study preferred a more passive role in the decision making process in the sense that respondents expressed a greater preference for their GPs to make all or most decisions. Despite evidence suggesting that active patient involvement in medical decision making may result in increased treatment adherence and improved outcomes [37], research on patient and provider characteristics associated with patient decision-making role preferences found that patients receiving lower levels of provider communication about decision making as well as patients being very satisfied with their care are more likely to prefer provider-made decisions [38]. Although GPs enjoyed a high degree of patients’ trust, on a daily basis they fell short of their expectations since they often provided minimal or no instructions when prescribing medication. Thus patients were left to their own devices to seek for information from sources that are more readily available then GPs; however, at times these were less reliable sources such as the internet [33].

Due to the fact that the focus groups were conducted in a German setting and participants made reference to PILs published in German, the transferability of the results presented in this study could be limited and they deserve further investigation. Besides, the various response behaviors described above were triggered in relation to PILs taken from medication commonly prescribed for the treatment of Type 2 diabetes, hypertension and hypercholesterolemia. Therefore it remains unclear what emotional reactions patients would have described if they were provided with PILs for other medications. Furthermore, the data of our study have been generated from a predominantly male sample aged 50 and above which could have influenced our findings. However, our study sample appears to be a typical representation of patients with Type 2 diabetes, hypertension and hypercholesterolemia who present at GP practices. Finally, although there were three patients involved in the focus groups with a migrant background, it was not our intention to look for differences between migrants and non-migrants.

## Conclusions

The findings of our qualitative study have shown that current PILs convey risk information in a way that provoked feelings of fear and anxiety in the reader. Such negative emotions cause patients to make alterations to their prescribed treatment regimen without prior consultation of their GPs. The raison d’être of PILs is to inform patients about application and risks of the prescribed medication in a clear, understandable and readily readable way. Yet, our results suggest that reading PILs is associated with a decrease in medication adherence. The challenge in designing future PILs is to develop presentations which allow the conveyance of risk information in a way that is perceived as less frightening by patients but will still provide vital information necessary to make an informed decision on whether or not to take the prescribed medication. An informed decision not to take a specific medicine is an acceptable outcome. From the patients’ point of view, spoken information provided by their GPs is preferred to reading PILs. This holds true especially for the conveyance of risk information. The quality and the extent of the patient-physician relationship may also contribute to calming down frightened patients. Yet physicians are also encouraged to welcome patients who make use of written information in order to evoke questions that can be discussed during consultation with their GPs.

To improve PILs further it is suggested that regulators and producers of such written information consider greater involvement of target patient groups at all stages of the production process. More precisely, during the production process specific emphasis should be placed on testing the degree of emotional arousal provoked in patients when reading certain risk information. Future generations of PILs should aim at providing information about possible side effects and drug interactions in a language of risk that is causing less anxiety in order to diminish or – if at all possible – avoid typical "knee-jerk reactions" such as altering the dosage or discontinuing the prescribed medication. This is vital because Hartley & Phelps ( [39]; p.8) conclude from their review on the relationship between anxiety and decision-making that:

"anxiety increases the attention to negative choice options, the likelihood that ambiguous options will be interpreted negatively and the tendency to avoid potential negative outcomes even at the cost of missing potential gains."

In order to improve the outcome of a benefit-risk-assessment of a medication the European Commission should consider including emotional arousal testing as an additional aspect in the guideline on how to produce package leaflets.

## References

[1] European Parliament and the Council of the European Union (2004). Directive 2001/83/EC of the European Parliament and of the Council of 6 November 2001 on the Community Code relating to medicinal products for human use.

[2] European Parliament and the Council of the European Union (2004). Amended by Directive 2004/27/EC of the European Parliament and of the Council of 31 March 2004 amending Directive 2001/83/EC on the Community code relating to medicinal products for human use.

[3] European Commission (2009). Guideline on the readability of the labelling and package leaflet of medicinal products for human use. Revision 1.

[4] Quality Review of Documents Group (2010). QRD Annotated Template: Revision of the Product Information.

[5] Quality Review of Documents (2009). Draft Version of the QRD Annotated Template for External Consultation.

[6] Maat HP, Lentz L (2010). Improving the usability of patient information leaflets. Patient Educ Couns.

[7] Carrigan N, Raynor DK, Knapp P (2008). Adequacy of patient information on adverse effects: an assessment of patient information leaflets in the UK. Drug Saf.

[8] Gigerenzer G, Edwards A (2003). Simple tools for understanding risks: from innumeracy to insight. Brit Med J.

[9] Nink K, Schröder H (2005). Zu Risiken und Nebenwirkungen: Lesen Sie die Packungsbeilage?.

[10] Hamrosi KK, Raynor DK, Aslani P (2013). Pharmacist, general practitioner and consumer use of written medicine information in Australia: are they on the same page?. Res Social Adm Pharm.

[11] McDonnell PJ, Jacobs MR (2002). Hospital admissions resulting from preventable adverse drug reactions. Ann Pharmacother.

[12] Rodgers PT, Ruffin DM (1998). Medication nonadherence: part II — a pilot study in patients with congestive heart failure. Manag Care Interface.

[13] Fuchs J, Hippius M (2007). Inappropriate dosage instructions in package inserts. Patient Educ Couns.

[14] Fuchs J, Heyer T, Langenhan D, Hippius M (2008). Influence of front sizes on the readability and comprehensibility of package inserts. Pharm Ind.

[15] Anlauf M, Schwabe U, Paffrath D (2006). Antihypertonika. Arzneiverordnungsreport.

[16] Robert Koch-Institut (2006). Gesundheit in Deutschland. Gesundheitsberichterstattung des Bundes.

[17] McLafferty I (2004). Focus group interviews as a data collecting strategy. J Adv Nurs.

[18] Halcomb EJ, Gholizadeh L, DiGiacomo M, Phillips J, Davidson PM (2007). Literature review: considerations in undertaking focus group research with culturally and linguistically diverse groups. J Clin Nurs.

[19] Morgan DL (1996). Focus groups. Annu Rev Sociol.

[20] Jayasekara RS (2012). Focus groups in nursing research: methodological perspectives. Nurs Outlook.

[21] Kruse J (2014). Qualitative Interviewforschung. Ein integrativer Ansatz.

[22] Creswell J (1998). Qualitative Inquiry and Research Design: Choosing Among Five Traditions.

[23] Kidd PS, Parshall MB (2000). Getting the focus and the group: enhancing analytical rigor in focus groups research. Qual Health Res.

[24] Ritchie J, Spencer L, Bryman A, Burgess R (1994). Qualitative data analysis for applied policy research. Analyzing Qualitative Data.

[25] Lincoln YS, Guba EG (1985). Naturalistic Inquiry.

[26] Koo M, Krass I, Aslani P (2002). Consumer opinions on medicines information and factors affecting its use - an Australian experience. Int J Pharm Pract.

[27] Lee DYL, Armour C, Krass I (2007). The development and evaluation of written medicines information for type 2 diabletes. Health Educ Res.

[28] Fuchs J, Götze EA (2009). Patientengerechte Arzneimittelinformation in Packungsbeilagen. Pharm Ind.

[29] Weitbrecht WU, Voßkämper C (2002). Influence of the drug package information paper on compliance of neurological and psychiatric outpatients. Fortschr Neurol Psychiatr.

[30] Clerehan R, Buchbinder R, Moodie J (2005). A linguistic framework for assessing the quality of written patient information: its use in assessing methotrexate information for rheumatoid arthritis. Health Educ Res.

[31] Gal I, Prigat A (2005). Why organizations continue to create patient information leaflets with readability and usability problems: an exploratory study. Health Educ Res.

[32] Vander Stichele RH, De Potter B, Vyncke P, Bogaert MG (1995). Attitude of physicians toward patient package inserts for medication information in Belgium. Patient Educ Couns.

[33] Edwards B, Chakraborty S (2012). Risk communication and the pharmaceutical industry. What is the reality?. Drug Saf.

[34] Davis JJ, Cross E, Crowley J (2007). Pharmaceutical websites and the communication of risk information. J Health Commun.

[35] Williams B, Brown D (2012). Direct to consumer internet advertising of statins: an assessment of safety. Pharmacoepidemiol Drug Saf.

[36] Nair K, Dolovich L, Cassels A, McCormack J, Levine M, Gray J, Mann K, Burns S (2002). What patients want to know about their medications. Focus group study of patient and clinician perspectives. Can Fam Physician.

[37] Fraenkel L, McGraw S (2007). What are the essential elements to enable patient participation in medical decision making?. J Gen Intern Med.

[38] Kumar R, Korthuis PT, Saha S, Chander G, Sharp V, Cohn J, Moore R, Beach MC (2010). Decision-making role preferences among patients with HIV: associations with patient and provider characteristics and communication behaviors. J Gen Intern Med.

[39] Hartley CA, Phelps EA (2012). Anxiety and decision-making. Biol Psychiatry.

[CR40] The pre-publication history for this paper can be accessed here:http://www.biomedcentral.com/1471-2296/15/163/prepub

